# Early gastric mixed neuroendocrine–non-neuroendocrine neoplasms with endoscopic findings of neuroendocrine cell carcinoma components exposed on the mucosal surface: a case report

**DOI:** 10.1186/s13256-022-03657-4

**Published:** 2022-11-14

**Authors:** Yohei Ito, Yoshiaki Kimoto, Rikimaru Sawada, Shinya Nagae, Koichi Furuta, Nao Takeuchi, Shunya Takayanagi, Yuki Kano, Rindo Ishii, Takashi Sakuno, Kohei Ono, Ryoju Negishi, Yohei Minato, Takashi Muramoto, Ken Ohata

**Affiliations:** Department of Gastroenterology, NTT Medical Center Tokyo, 5-9-22 Higashi-Gotanda, Shinagawa-Ku, Tokyo, 141-8625 Japan

**Keywords:** MiNENs, MANEC, ESD, Early gastric cancer

## Abstract

**Background:**

Gastric mixed neuroendocrine–non-neuroendocrine neoplasms are rare malignant tumors. The lack of specific findings makes it difficult to diagnose endoscopically. We report the case of early gastric mixed neuroendocrine–non-neuroendocrine neoplasms treated by endoscopic submucosal dissection.

**Case presentation:**

An 81-year-old Japanese female underwent esophagogastroduodenoscopy for screening and was treated with endoscopic submucosal dissection for the diagnosis of early gastric cancer. Histopathologically, the lesion was diagnosed as mixed neuroendocrine–non-neuroendocrine neoplasms (tubular adenocarcinoma 2 60%, endocrine cell carcinoma 40%), pT1b(submucosa (SM) 900 μm), pUL(−), Ly(+), v(−), pHM0, pVM0. After additional surgical resection without adjuvant chemotherapy, she has had no recurrences or metastases for 3 years.

**Conclusions:**

Comparing narrow-band imaging magnified endoscopic findings with pathological findings, the depressed area with a lack of surface structure was consistent with the neuroendocrine cell carcinoma component, while narrow-band imaging magnification findings showed non-network vessels. In this case, we examined endoscopic findings of early stage mixed neuroendocrine—non-neuroendocrine neoplasms in detail and compared it with the pathological findings. We believe that these endoscopic findings contribute to the diagnosis of mixed neuroendocrine–non-neuroendocrine neoplasms and can lead to its early detection.

## Background

Mixed neuroendocrine–non-neuroendocrine neoplasms (MiNENs) are rare neoplasms defined as mixed epithelial neoplasms composed of both neuroendocrine and non-neuroendocrine components, accounting for at least 30% of the neoplasm each [1, 2].

MiNENs have been identified in various organs, including the pancreas, colon, biliary tract, and stomach. In general, they are associated with aggressive behavior, strong invasiveness, and high lymph node dissemination [3]. It has been suggested that MiNENs patients may have worse prognosis compared with those with isolated gastric adenocarcinoma and neuroendocrine carcinoma [4, 5].

Therefore, early diagnosis and treatment are important. However, specific endoscopic findings are not well known, and many cases are discovered in advanced stages of cancer. In this study, we report the results of endoscopic studies of an early stage of MiNENs detection, which confirmed the usefulness of endoscopic findings for diagnosis by comparing endoscopic findings with pathological findings.

## Case presentation

An 81-year-old Japanese woman was referred to our hospital for endoscopic treatment under the diagnosis of early stage gastric cancer (biopsy tub1–2) based on esophagogastroduodenoscopy (EGD) screening. She had no history of illness, and biochemical parameters and tumor markers were within normal limits.

*Helicobacter pylori* antibodies were negative, and EGD showed atrophic changes associated with previous *H. pylori* infection.

**Fig. 1:**
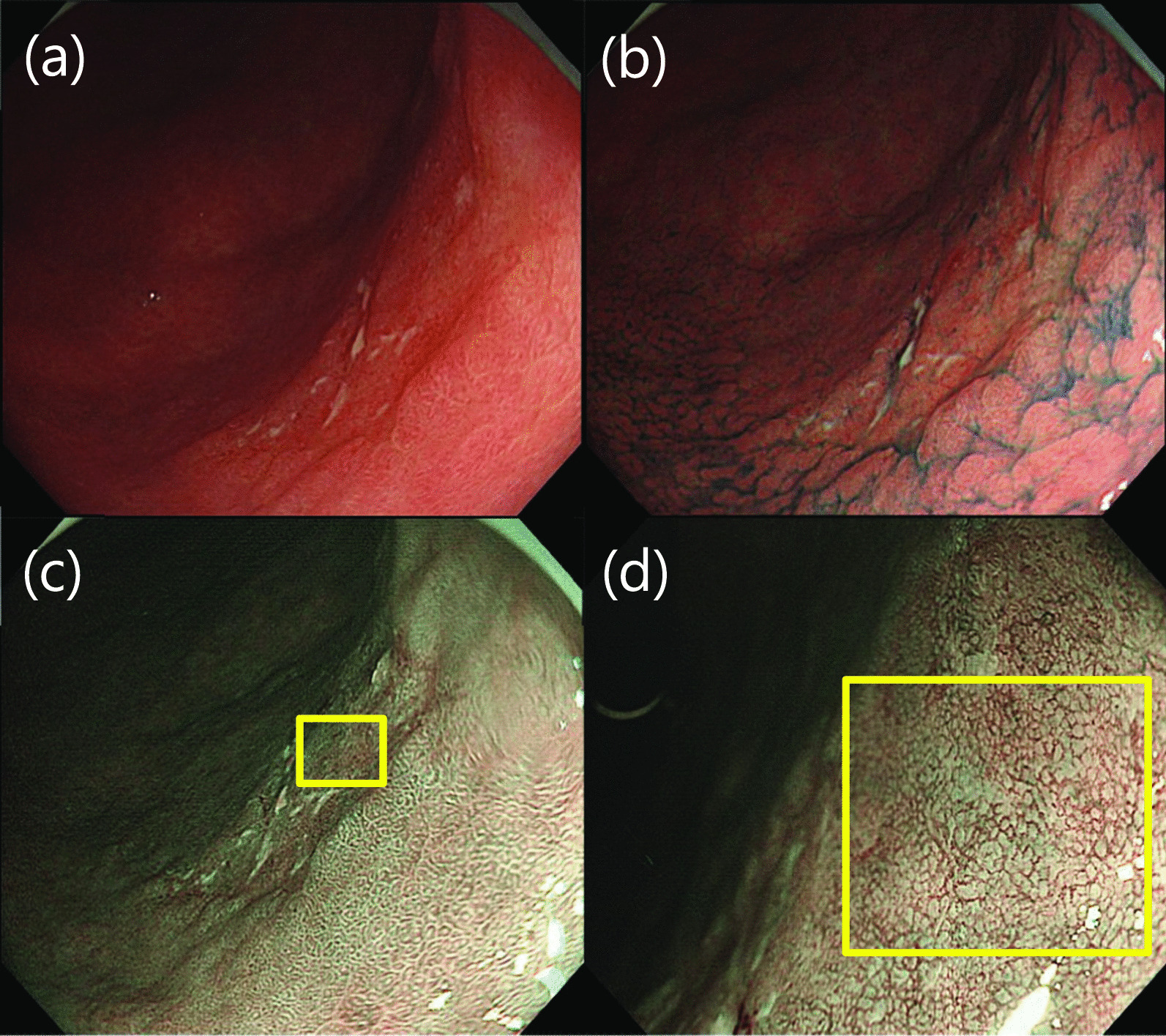
EGD findings. **a**, **b** An irregular depressed lesion on the posterior wall of the lower body. **c** NBI of depression lesion. **d** Magnifying endoscopy with NBI of the area circled by yellow square

**Fig. 2:**
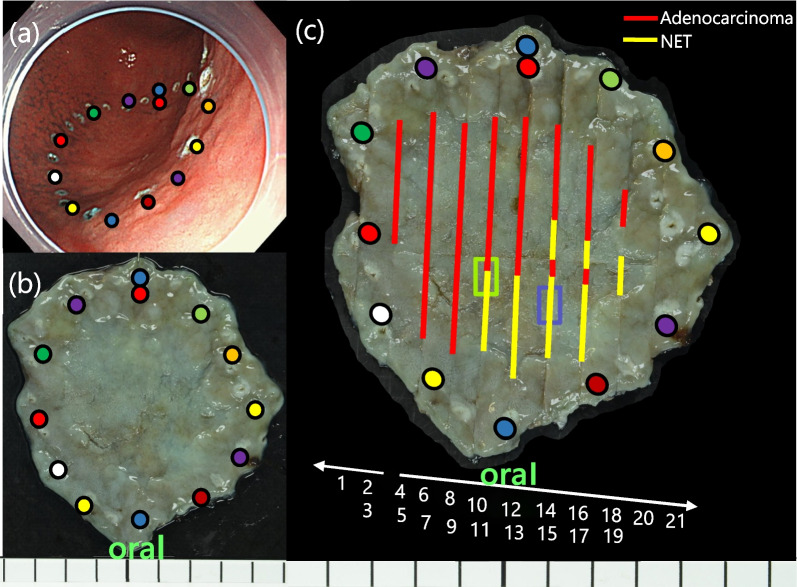
ESD findings. **a** Markings were placed around the lesion and endoscopic submucosal dissection was performed. **b** The macroscopic finding of the specimens resected by endoscopic submucosal dissection. **c** The pathological diagnosis was early gastric MiNENs

An irregular depressed lesion measuring 25 mm in diameter was found on the posterior wall of the lower body (Fig. 1a, b). Narrow-band imaging (NBI) revealed a demarcation line consistent with the surface of the depression (Fig. 1c). The surface structure within the depression was obscured, and the vessels were of variable caliber, with a network or collapsed network structure (Fig. 1d). Based on these findings, the patient was diagnosed with a well-differentiated intramucosal adenocarcinoma [tubular adenocarcinoma (tub1–2)], and endoscopic submucosal dissection (ESD) was performed (Fig. 2a). The resected specimen had the following histological parameters: 28 × 28 mm, type 0–IIc, MiNEN (tub2 60% > endocrine cell carcinoma 40%), pT1b(submucosa (SM) 900 μm), pUL(−), Ly(+), v(−), pHM0, pVM0 (Fig. 2b). Histologically, the tumor was composed of two components [tub2 adenocarcinoma and neuroendocrine cell carcinoma (NEC)], and it showed submucosal and lymphatic invasion. As shown in Fig. 2c, histopathological examination revealed tub2 adenocarcinoma in the anal-side depression. In the oral-side depression, tumor cells lacked glandular structures and had nested trabecular or acinar-like structures. Immunohistochemistry was positive for synaptophysin and CD56, and chromogranin partially, confirming the diagnosis of NEC. The final diagnosis was early gastric MiNEN as the adenocarcinoma and NEC components independently accounted for > 30% of the entire lesions. We determined that additional surgical resection was necessary due to submucosal and lymphatic invasion and laparoscopic pyloric gastrectomy was performed. The resected specimen showed no histological remnant of cancer and no metastasis in all the dissected lymph nodes. No postoperative adjuvant chemotherapy was administered, but annual computed tomography (CT) and EGD follow-up have been performed for 3 years without any recurrence or metastasis.

## Discussion and conclusions

The pathogenesis of MiNENs is as follows: (1) some cells acquire endocrine differentiation in adenocarcinoma tissue; (2) it is derived from pluripotent cells that have the ability to differentiate into both endocrine and exocrine differentiation; and (3) endocrine and exocrine tumors arise in the same site (collision carcinoma) [6, 7]. In the present case, the adenocarcinoma component of MiNENs and the NEC component were partially and irregularly intermingled, and each component was distinguishable immunohistologically as glandular and endocrine, suggesting that the adenocarcinoma had dedifferentiated and developed into a neuroendocrine carcinoma. Recently, it has been reported that gastrointestinal MiNENs, along with gastrointestinal NEC, share common molecular biological characteristics with the NEC component of adenocarcinoma coexisting in the same case, suggesting the involvement of molecules common to adenocarcinoma as well as NEC in the mechanism of MiNENs development.

**Fig. 3:**
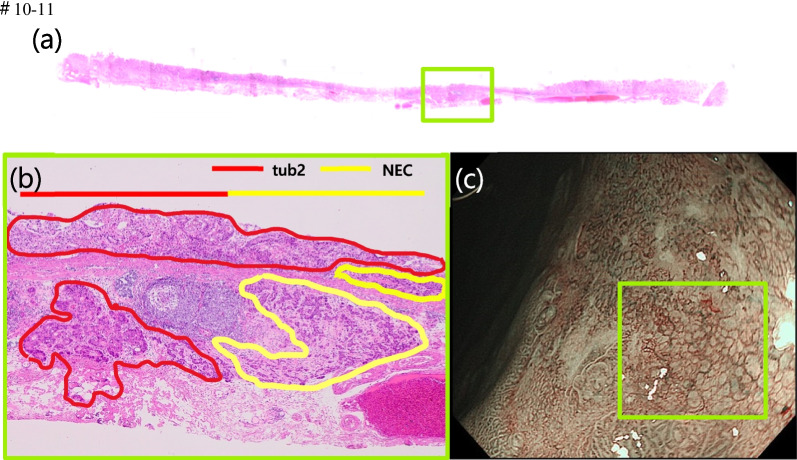
Comparison of histopathological findings with endoscopic findings. **a** Loupe images of the yellow-green square of the resected specimen. **b** The pathological diagnosis of the resected lesions was moderate-differentiated adenocarcinoma in the area circled in red and NEC in the area circled in yellow. **c** NBI magnification findings indicate the tub2 component of the mucosal surface layer

The NEC component is often localized in the deepest part of the tumor, as in the present case (Fig. 3b), and when it proliferates, it presents a submucosal tumor-like morphology. As the tumor progresses further, it is reported that the normal adenocarcinoma on the mucosal surface is shed, and the center of the tumor gradually becomes concave and ulcerated.

It is considered that the NEC component has more rapid growth and invasive ability than the adenocarcinoma component, resulting in the loss of the normal adenocarcinoma in the mucosal surface layer.

**Fig. 4:**
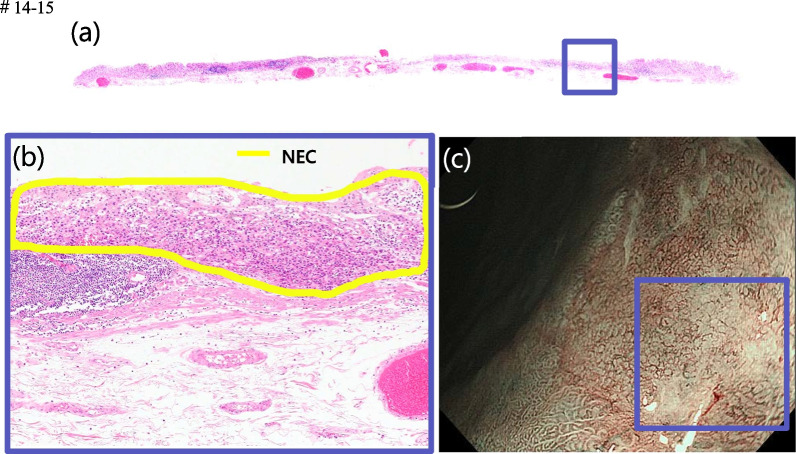
Comparison of histopathological findings with endoscopic findings. **a** Loupe images of the blue square of the resected specimen. **b** The pathological diagnosis of the resected lesions was NEC in the area circled in yellow. **c** NBI magnification findings indicate the NEC component of the mucosal surface layer

In this case, NBI magnification revealed that most of the depressed surface of the lesion showed surface structures and vascular structures suspicious for histological type tub1–2, but as shown in Fig. 4c, some of the surface structures disappeared at the mouth side of the depressed surface, and non-network vessels with dilated, tortuous, irregular caliber and uneven shape were observed. Comparing the NBI magnified endoscopic findings with the pathological findings, the NEC component was observed almost coincidentally with the area where the surface structure inside the depression disappeared and non-network vessels were observed in the NBI magnified findings. Therefore, a precise biopsy of the area with no structure and non-network vessels is important for diagnosis.

Although the magnified endoscopic findings of NEC suggest irregularities or loss of surface structure in the tumor area [9], there have been no detailed reports comparing the magnified endoscopic findings with the pathological findings. In the present study, we examined the preoperative endoscopic findings of early stage MINEN in detail and compared them with the pathological findings. We found that there were unstructured and non-networked vessels in the NEC-exposed areas. Furthermore, the NBI-magnified findings in Fig. 3c are suggestive of differentiated adenocarcinoma because the network structure is preserved but the size of the network is larger than that of other NBI magnified findings (Fig. 1d). The presence of the NEC component in the deeper layers results in the stretching and enlargement of the network structure in the superficial layers. In other words, the larger size of the network structure also suggests the presence of NEC in the deeper layers. However, it is difficult to distinguish between these findings and those of non-structural and non-network vessels, as they are similar to those seen in poorly differentiated adenocarcinoma. In cases such as the present case, in which the surface structure within the depression was lost because it was surrounded by highly differentiated adenocarcinoma, it is important to consider not only the presence of poorly differentiated adenocarcinoma, but also the presence of NEC, and to perform immunostaining aggressively. In the present case, there was no submucosal tumor-like growth, and endoscopic ultrasonography (EUS) was not performed. However, since NEC components are often detected by submucosal invasion, EUS may be effective in diagnosing the depth of NEC if NEC can be identified by NBI magnification findings.

We believe that MiNEN is a tumor that requires further histopathological and clinical investigation as more cases are accumulated in the future.

## Data Availability

The datasets used and/or analyzed during the current study are available from the corresponding author on reasonable request.

## Abbreviations

MiNENs: Mixed neuroendocrine–non-neuroendocrine neoplasms
NEC: Neuroendocrine cell carcinoma
ESD: Endoscopic submucosal dissection
NBI: Narrow-band imaging
EGD: Esophagogastroduodenoscopy
EUS: Endoscopic ultrasonography
